# Development of deep learning-based novel auto-segmentation for the prostatic urethra on planning CT images for prostate cancer radiotherapy

**DOI:** 10.1007/s12194-024-00832-8

**Published:** 2024-08-14

**Authors:** Hisamichi Takagi, Ken Takeda, Noriyuki Kadoya, Koki Inoue, Shiki Endo, Noriyoshi Takahashi, Takaya Yamamoto, Rei Umezawa, Keiichi Jingu

**Affiliations:** 1 Course of Radiological Technology, Health Sciences, Tohoku University Graduate School of Medicine, 2-1, Seiryo-machi, Aoba-ku, Sendai, 980-8575 Japan; 2 Department of Radiation Oncology, Tohoku University Graduate School of Medicine, Sendai, Japan; 3 Elith Inc., Shibuya, Tokyo Japan

**Keywords:** Prostatic urethra, Urinary toxicity, Prostate cancer, Deep learning technology, CT image

## Abstract

Urinary toxicities are one of the serious complications of radiotherapy for prostate cancer, and dose-volume histogram of prostatic urethra has been associated with such toxicities in previous reports. Previous research has focused on estimating the prostatic urethra, which is difficult to delineate in CT images; however, these studies, which are limited in number, mainly focused on cases undergoing brachytherapy uses low-dose-rate sources and do not involve external beam radiation therapy (EBRT). In this study, we aimed to develop a deep learning-based method of determining the position of the prostatic urethra in patients eligible for EBRT. We used contour data from 430 patients with localized prostate cancer. In all cases, a urethral catheter was placed when planning CT to identify the prostatic urethra. We used 2D and 3D U-Net segmentation models. The input images included the bladder and prostate, while the output images focused on the prostatic urethra. The 2D model determined the prostate’s position based on results from both coronal and sagittal directions. Evaluation metrics included the average distance between centerlines. The average centerline distances for the 2D and 3D models were 2.07 ± 0.87 mm and 2.05 ± 0.92 mm, respectively. Increasing the number of cases while maintaining equivalent accuracy as we did in this study suggests the potential for high generalization performance and the feasibility of using deep learning technology for estimating the position of the prostatic urethra.

## Introduction

Prostate cancer is the most frequent cancer among Japanese males, and the number of patients with the condition in Japan is increasing every year [1]. Prostate cancer has a relatively good prognosis, with the 5 year relative survival rate for localized prostate cancer in Japan being reported to be as high as 100% and the net survival rate for prostate cancer as high as 95.1% [1]. Therefore, not only treatment outcomes but also post-treatment quality of life (QOL) have improved, leading to research focusing on QOL and its improvement [2–4].

For prostate cancer, radiotherapy is one treatment that offers better preservation of function compared to surgery [5–7]. Dose escalation has been reported for radiotherapy for localized prostate cancer, and dose administration using highly accurate radiotherapy techniques such as intensity-modulated radiotherapy (IMRT) has been reported to improve treatment outcomes while reducing adverse events in the rectum and bladder [8, 9]. However, urinary toxicity is a serious complication of radiotherapy for prostate cancer [10], and it continues to be a persistent issue. There have been studies investigating the relationship between the prostatic urethra and urinary disorders, and Takeda et al. reported that late urinary toxicity may develop more than 10 years after treatment [11]. Urinary toxicity significantly affects patients’ QOL [12], and Åkerla et al. reported that patients with urinary toxicity, regardless of whether or not they received radiotherapy, have a higher mortality rate than those without urinary toxicity [13]. One reason for the persistence of urinary disorders is the difficulty in visualizing the prostatic urethra in conventional planning CT (pCT). Visualization on pCT may require a urethral catheter; however, it would be an invasive procedure for patients, which results in increased patient burden (although temporary).

In recent years, the application of deep learning in the medical field has advanced, with various studies being performed on image reconstruction [14], prognosis prediction [15], diagnosis, and CAD systems [16]. Organ segmentation in medical imaging is one example of deep learning [17], with several methods achieving a clinical usability benchmarking Dice coefficient of 0.8 in pelvic segmentation [18, 19].

Some studies aim to identify the position of the prostatic urethra without using a urethral catheter. Acosta et al. and our laboratory [20, 21] developed a multi atlas-based technique for estimating the position of the prostatic urethra, reporting mean centerlines distances (CLDs) of 2.77 mm and 2.09 mm, respectively. These studies attempted to estimate the prostatic urethra from the contours of the bladder and prostate, and suggested that these contours might contain information regarding the location of the prostatic urethra. Studies using deep learning have also been conducted. Belue et al. [22] developed a technique using deep learning on MRI images and reported a CLD of 2.59 mm. In addition, Cubero et al. [23] reported a CLD of 1.6 mm in a study conducted using CT images and deep learning-rendered data of surrounding organs. However, Cubero’s study was conducted on patients undergoing brachytherapy using low-dose-rate sources (LDR-BT), and there is a paucity of studies employing deep learning applicable to patients receiving external beam radiation therapy (EBRT). In addition, studies on CT images have been conducted on only a few cases, especially with the number of test cases being limited to 11–20. Our multi atlas-based method [21] inherently requires deformable image registration (DIR) operations among all cases to create training data, which involves significant computational time and labor costs for adding cases. Further DIR between CT and MRI is necessary, and the use of DIR across different modalities can decrease accuracy [24]. To solve such problems, we have significantly increased the number of cases treated with curative IMRT planning using a urethral catheter upon pCT. This study aimed to develop a deep learning-based technique for estimating the position of the prostatic urethra and evaluate its accuracy.

## Materials and methods

### Data

In this study, we examined 430 patients with localized prostate cancer who underwent curative IMRT at Tohoku University Hospital between May 2005 and February 2019. All cases had contour information for the prostate, bladder, and prostatic urethra. These pCT images were obtained using a GE Light Speed RT16 (GE Medical Systems, Waukesha, WI, USA). The acquisition parameters were 120 kV and 500 mA, with a pixel size of 0.8–1.2 mm/px and slice thickness of 2.0 mm or 2.5 mm. The prostatic urethra is difficult to visualize in CT images; therefore, a urethral catheter had to be in place before its position could be determined via imaging. Contour creation and treatment planning were conducted using Eclipse (Ver 8.6 or Ver 13.2, Varian Medical Systems, Palo Alto, CA, USA). Contouring was performed by experienced radiation oncologists.

### Workflow

**Fig. 1:**
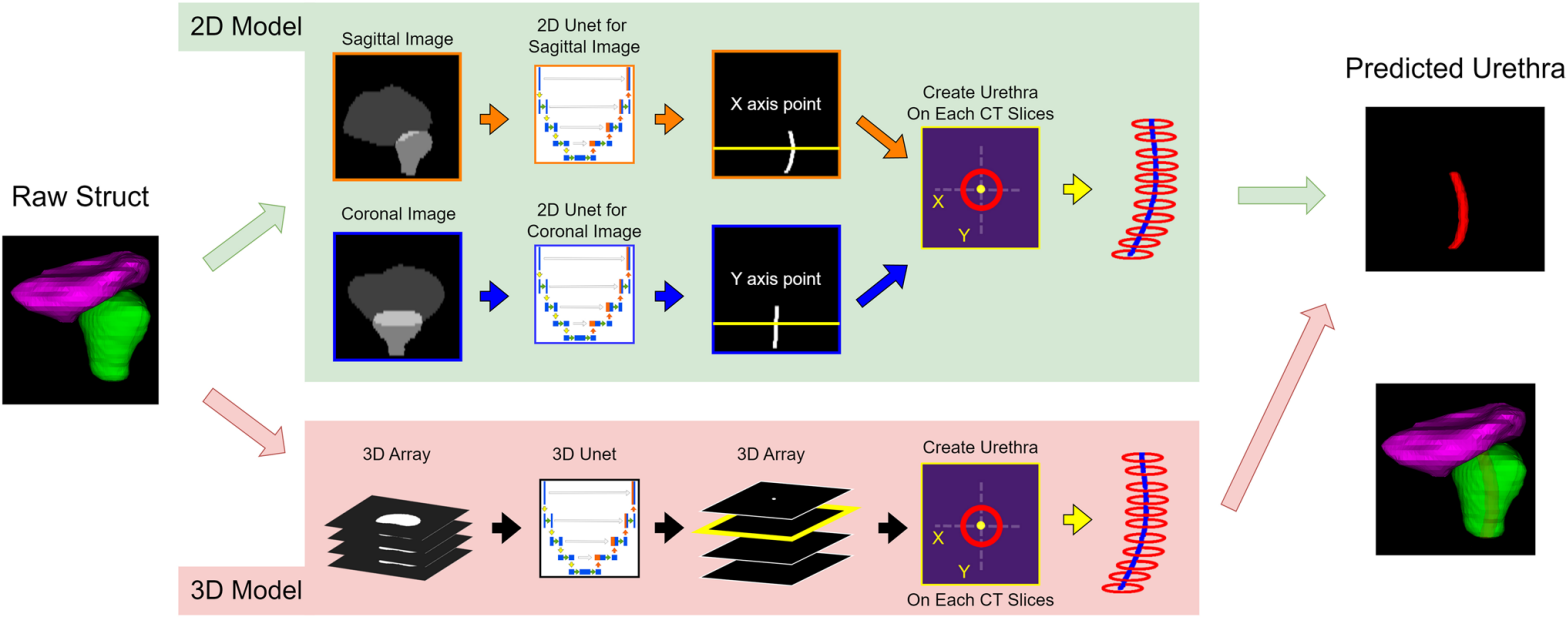
Workflow of our methods. We used 2D or 3D U-Net to estimate the prostatic urethra. Input data for deep learning model are the gray-scaled bladder and prostate. Two-dimensional data were created by projecting 3D data in the coronal and sagittal directions. Output from deep learning models could be irregularly shaped; therefore, the coordinates of the prostatic urethra were computed on each CT slice and a circle using the coordinates was created

Figure 1 shows the workflow for this method. In this study, we built a prostatic urethra estimation system using 2D or 3D U-Net segmentation models.

### Data creation

For the deep learning model inputs and outputs, 256-level grayscale 2D or 3D data created from contour information were used. The data did not include the CT images themselves and only contour information. This was because the CT images showed the urethral catheter, and the study aimed to apply the model to cases where catheter was absent. All the patients enrolled in this study had a urethral catheter inserted, however, we conducted the study simulating data without the catheter. The input data were created from the bladder and prostate contours, and the output data were created from the prostatic urethra that had been inserted through the urinary catheter. DICOM RT Structure Set data were converted to separate binary images for each contour using 3D Slicer V5.0.3 (www.slicer.com) [25]. Furthermore, the binarized data for each organ was edited to create grayscale images using Python 3.8.10 code (Python Software Foundation, Wilmington, DE, USA). All binary data were resampled to a voxel size of 1 mm × 1 mm × 1 mm using cubic spline interpolation. Subsequently, the input data were created from the resampled binarized data of the bladder and prostate, and the brightness values were set to 64 and 128 for the bladder and the prostate, respectively. The output data were created from the resampled binarized data of the prostatic urethra, and the brightness value was set to 255. The 2D data were created by projecting 3D data in the coronal and sagittal directions. To crop these data, the prostate was centered at the bottom of the image, and the image size was set to 128 × 128 × 128 for 3D data and 256 × 256 (2 × high resolution from original data) for 2D data.

### Model structure/learning

We used 2D or 3D U-Net as the model structure to compare their performance. 2D U-Net has a lower computational cost, and 3D U-Net can utilize features of the input data directly. All models were implemented with the Medical Open Network for Artificial Intelligence (https://monai.io/), which is a Pytorch-based deep learning framework. The model structures are presented in Table 1. Of the 430 cases, 70% were used for training, 10% for validation, and 20% for testing. They were divided randomly, and the division was the same for both 2D and 3D. To ensure a generalization performance, the following data augmentations were randomly applied during training: random scaling, random crop, random rotation, translation, and random elastic deformation. The evaluation and loss functions were performed using Dice Cross Entropy Loss (DiceCELoss). The optimizer was Adam, with a learning rate of 0.0001. To determine the training conditions, we performed fivefold cross-validation within only the training cases, adopting the one with the lowest DiceCELoss. The determined learning conditions are also presented in Table 1. We then trained other models with established conditions using training and validation data as the final model. The early stop function was used, and the model was adopted when the loss on validation data was smallest.

**Table 1:** Model structure and learning conditions

	2D	3D
Model Settings		
Number of residual units	3	4
Channels per units	16, 32, 64, 128, 256	32, 64, 128, 256, 512
Strides	2	2
Dropout	0.5	0
Training conditions		
Loss function	Dice CE loss	Dice CE loss
Optimizer	Adam	Adam
Learning rate	0.0001	0.0001
Batch size	4	2
Epochs	1200	1000

### Urethra creation

In this study, instead of using the model’s output directly as the urethra, a circle was drawn by calculating coordinates from the prediction results. The calculation of coordinates was performed at positions corresponding to resampled slices (X–Y planes). In the 2D model, the X coordinate was identified from the coronal images, and the Y coordinate from the sagittal images. In the 3D model, center of the prediction result was identified on the X–Y plane, and these were considered as the X and Y coordinates of the prediction result. This process was applied to X–Y planes in which the prostate was present, and the resulting cylindrical region with a diameter of 5 mm was defined as the prostatic urethra. Finally, they were resampled to the original pixel size of the CT using cubic spline interpolation.

### Evaluation

The urethra position in this study was evaluated using the Average Centerline Distance (CLD) and the percentage of segmented centerline lying within a radius (PWR) for each patient. The CLD was computed from the distance between the predicted and ground truth midpoints for each CT slice and averaged over these values. The CLD was computed for the upper, middle, and lower thirds of the prostate, as well as for the entire region. The PWR represents the proportion of predicted centerline segments within a certain distance from the ground truth. Similar to the CLD, it was determined for each CT slice, calculated for both 3.5 mm and 5 mm in this study. Centerlines of the predicted results and ground truth were created by calculating the centroids of the contours for each CT slice. The CLD and PWR were calculated using an in-house Python code.

## Results

### Positional error

**Fig. 2:**
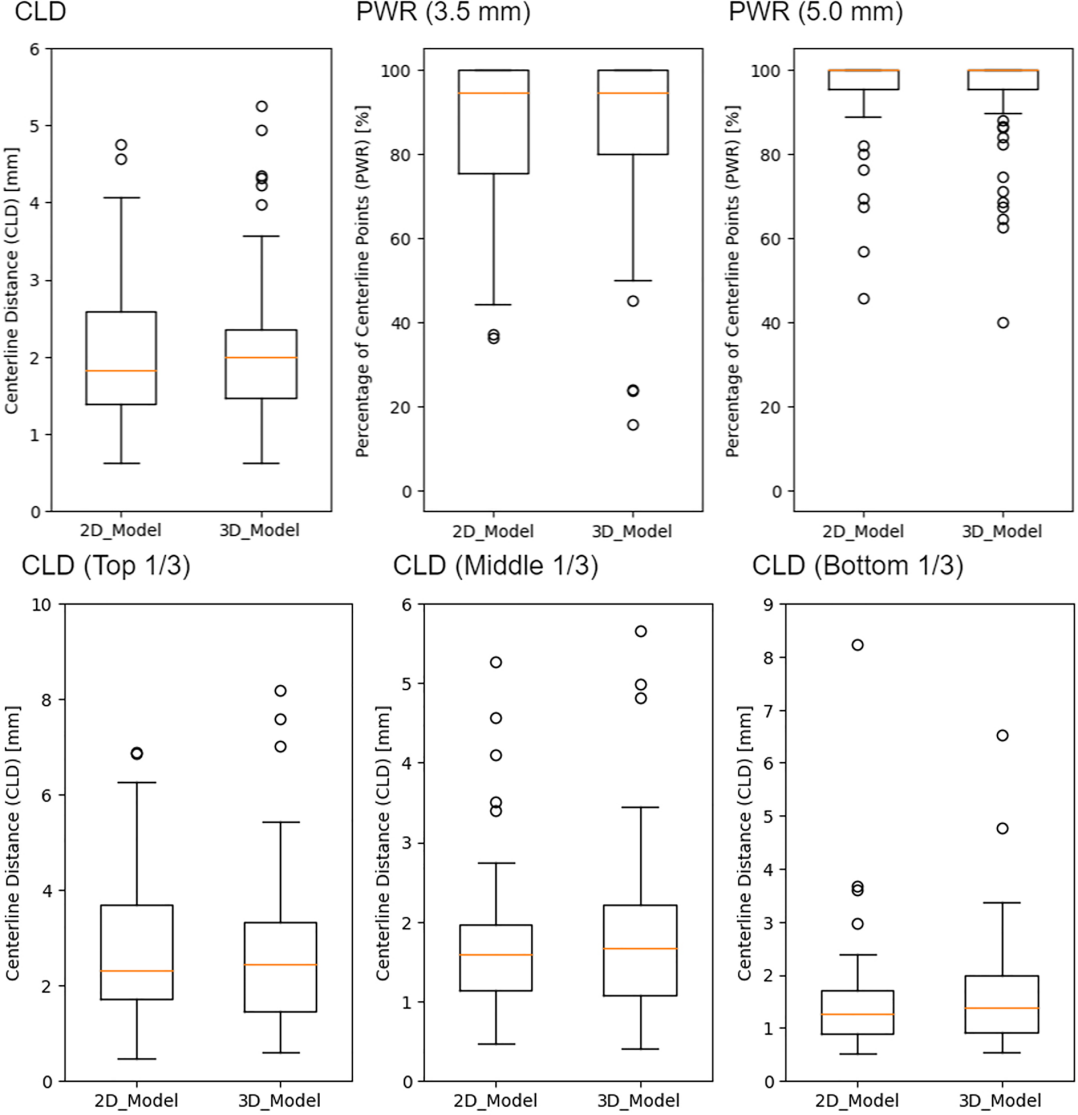
Box-and-whisker plots for the average centerline distance (CLD) and the percentage of segmented centerline lying within a radius (PWR). CLD is the average distance of the predicted urethra and ground truth obtained by the urinary catheter. It was calculated for the whole urethra and the top 1/3, middle 1/3, and bottom 1/3 of the prostate. The PWR is the percentage of sections where the difference is within a certain distance. We calculated the PWR for 3.5 mm and 5 mm

Box-and-whisker plots for CLD and PWR in that study are shown in Fig. 2. Average error in the 2D U-Net for the overall section was 2.07 ± 0.87 mm, and error in the 3D U-Net was 2.05 ± 0.92 mm. There were 42 cases in which the 2D model was more accurate and 45 cases in which the 3D model was more accurate, which showed roughly equivalent accuracy between the two models (*p* = 0.834). The accuracy difference between the two models was > 1 mm in 13 cases and > 2 mm in two cases. CLDs for upper, middle, and lower sections in the 2D and 3D models are presented in Table 2. In both of these models, lower section revealed higher accuracy and upper section exhibited lower accuracy. PWR are presented in Table 2 as well. The PWR results show that over 85% of the predicted urethra sections were within an error of less than 3.5 mm, and over 94% of the sections were within an error of less than 5 mm.

**Table 2:** Comparison with a previous study

	CLD (mm)				PWR(%)	
	Whole	Top 1/3	Mid 1/3	Bottom 1/3	3.5 mm	5.0 mm
2D Unet	2.07 ± 0.87	2.75 ± 1.71	1.81 ± 1.04	1.68 ± 1.17	85.85 ± 17.60	94.77 ± 11.91
3D Unet	2.05 ± 0.95	2.80 ± 1.81	1.77 ± 0.94	1.59 ± 0.94	86.63 ± 18.45	95.27 ± 10.36
Cubero et al	1.6 ± 0.8	1.7 ± 1.4	1.5 ± 0.9	1.7 ± 0.9	93.6 ± 12.4	97.4 ± 8.2
Belue et al	2.6 ± 1.3	3.7 ± 2.6	2.3 ± 1.4	1.5 ± 1.0	N/A	N/A
Takagi et al	2.06 ± 0.86	2.49 ± 1.78	1.86 ± 0.89	1.92 ± 0.94	N/A	N/A

The study by Cubero et al. was a deep learning-based method for CT images of patients with brachytherapy uses low-dose-rate sources, the study by Beule et al. was a deep learning-based method for MRI images, and the study by Takagi et al. was a multi atlas–based method with 120 patients (20 patients for test data)

*CLD* the centerlines distances, *PWR* the percentage of segmented centerline lying within a radius

**Fig. 3:**
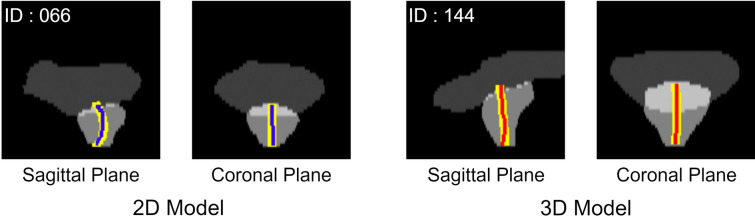
Case of best prediction. Yellow lines show the ground truth, the blue line shows the prediction from the 2D model, and the red line shows the prediction from the 3D model. Various cases show the best prediction for the 2D and 3D models

**Fig. 4:**
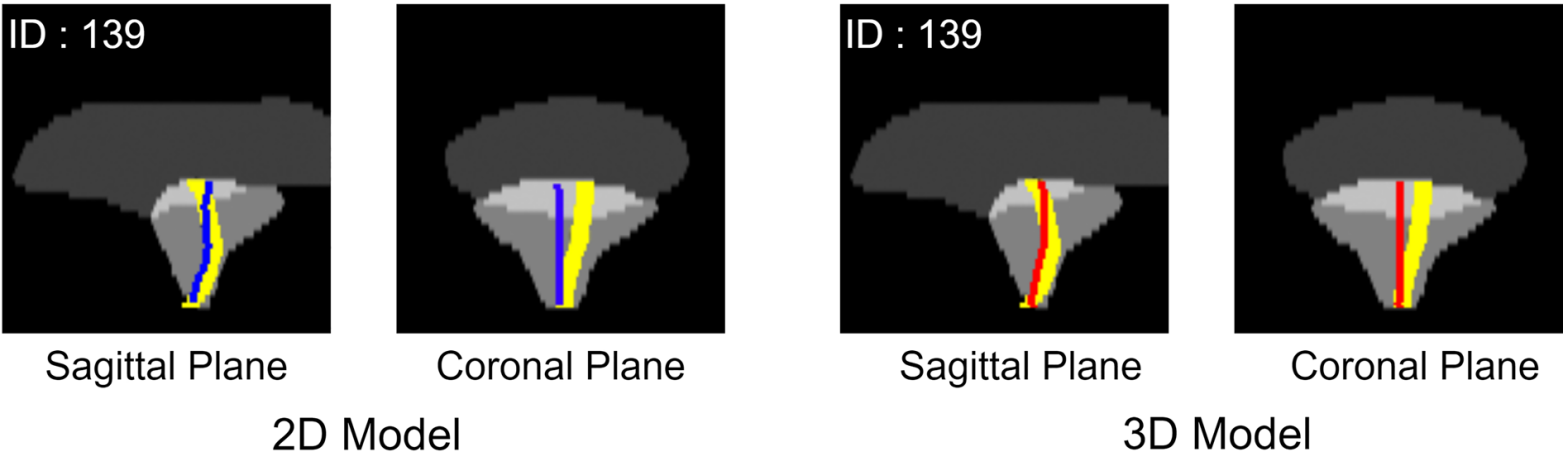
Case of worst prediction. Yellow lines show the ground truth, the blue line shows the prediction from the 2D model, and the red line shows the prediction from the 3D model. The same case shows the worst prediction for the 2D and 3D models. The CLD was 4.75 mm and 5.26 mm for the 2D and 3D models, respectively. Both these models could not predict the asymmetrical prostatic urethra and resulted in poor accuracy

Figures 3, 4 illustrate the cases with the highest and lowest accuracy for both models. The maximum error was 4.75 mm and 5.26 mm for the 2D model and 3D models (same case), respectively, and the minimum error was 0.58 mm and 0.63 mm for the 2D and 3D models (different case), respectively.

## Discussion

The present study attempted to estimate the intraprostatic urethra using a deep learning model on pCT images of IMRT cases. We used 2D and 3D U-Net models to estimate the position of the prostatic urethra, and the mean errors for each model were 2.07 ± 0.87 mm and 2.05 ± 0.92 mm, respectively, which indicates roughly equivalent accuracy. In the site-specific evaluation, both models showed the highest accuracy in the lower prostate and the lowest accuracy in the upper prostate. This is likely because the position where the prostate connects to the bladder at the top varies most between individuals, whereas the bottom part generally traverses the central part of the prostate. As depicted by the figures, tilting of the prostatic urethra in the left–right direction mostly occurred in the upper part of the prostate, likely contributing to worse CLD in the upper part of the prostate. The reason for the case with maximum error exhibited tilting in the left–right direction, suggesting that accuracy might be improved by incorporating factors such as benign prostatic hyperplasia that might cause left–right tilting.

The results of this study and those of previous studies are presented in Table 2. Similar trends were observed in other preceding studies using deep learning. The studies by Cubero et al. [23] on CT and automatically contoured OARs and by Belue et al. [22] on MRI images and our previous multi atlas-based method [21] were compared. Cubero et al. reported an average error of 1.6 ± 0.8 mm [23], which is higher in accuracy than that of our proposed method. Two possible reasons for this difference are considered. First, their study was limited to patients undergoing LDR-BT, which might have different characteristics than the IMRT patients used in our method. For example, the mean prostate volume of the patients used by Cubero et al. was 62.58 ± 15 mL, and in the data we used, the mean prostate volume of 35.67 ± 14.21 mL (14.59–100.60 mL). In addition, their study included 55 cases including training data and only 11 test cases, which might have affected the results. Second, their method used automatically contoured OARs to draw the prostatic urethra. They reported that the Dice coefficients of their automatic contouring model for the prostate and bladder were 93.6% and 83.3%, respectively, which could have affected the accuracy of the prostatic urethra.

Belue et al. reported an average accuracy of 2.56 mm for segmentation on MRI images [22]. Our study surpassed this accuracy, which may be attributed to the segmentation task using MRI images as input being significantly different from our method. In addition, the comparison of prostate regions (upper, middle, lower) yielded similar results.

Compared with our multi atlas–based method proposed in 2020, the results were equivalent. The overall CLD accuracy was almost the same, with slightly improved accuracy in the upper CLD. The test data in this study were more than four times larger, which might indicate that our dataset could include a wider variety of cases. This suggests that our method could be a more versatile and robust prediction model. In the previous multi atlas-based method, increasing atlas data requires executing DIR for all cases to create teacher data, which means the computation time quadratically increases with the number of cases, limiting the increase in atlas data. Deep learning-based methods also require more training time proportional to the teacher data; however, even with the number of cases in this study, training was completed within approximately 6 h for both the sagittal and coronal 2D models and within 24 h for the 3D model, making case addition easier. In addition, the multi atlas-based method introduced uncertainty due to atlas selection, consequently affecting its accuracy. This method utilized a machine learning (ML) model to select suitable atlases from a dataset. However, the ML model we developed was unable to consistently select the best atlases. Conversely, the deep learning-based method allowed us to bypass the need for atlas selection entirely, as it incorporated all training data directly into the model. From these aspects, deep learning-based methods may have advantages over multi atlas-based methods. In addition, the multi atlas-based method performs DIR between CTs; however, applications to increasingly used MRI images will involve CT-MRI DIR, risking reduced DIR accuracy [26–28]. Our method, which uses only contour information, does not have this problem and may be advantageous for combined modality use, including MRI-Linac.

Nevertheless, this study had some limitations. First, the ground truth of the prostatic urethra was obtained through the catheter, possibly including errors compared to cases in which there was no catheter. Dekura et al. [29] reported the possibility of the prostatic urethra being deformed by a urethral catheter, reporting an average error of 2.0 mm (0.3–7.6 mm). This error might have been included in the model we constructed. This issue is difficult to resolve in CT-based studies because of the invisibility of the prostatic urethra; however, our method, which uses only contour information, could be resolved using images obtained from methods such as MRI that often can visualize the prostatic urethra. It suggests that including the urethra estimated without a catheter in the training data might achieve urethra positioning accuracies comparable to those with the positional error caused by the catheter. Second, there are still cases that cannot be addressed. As in previous multi atlas-based methods and previous studies, cases where the true urethra tilts left and right due to prostate hypertrophy tend to have reduced accuracy, which was also observed in our method. This is likely because there were only a few cases with an asymmetrical prostatic urethra in the training data, and further generalization performance improvement requires an increase in the number of irregular cases such as those with asymmetrical prostatic urethras due to prostate enlargement. Therefore, some cases may have significant errors that potentially hinder clinical use.

## Conclusion

In this study, we investigated the estimation of the prostatic urethra’s position using deep learning for cases eligible for EBRT. Regardless of significantly increasing the test data compared to previous methods, results showed equivalent outcomes, suggesting their potential applicability to many cases. Furthermore, the ease of adding cases and extending to other modalities suggested the potential for clinical use.

## Data Availability

The data supporting the findings of this study are unavailable for public release, as consent has only been obtained for research use.
